# Aclarubicin: contemporary insights into its mechanism of action, toxicity, pharmacokinetics, and clinical standing

**DOI:** 10.1007/s00280-024-04693-1

**Published:** 2024-07-04

**Authors:** Aleksandra Murzyn, Justyna Orzeł, Natalia Obajtek, Anna Mróz, Dominika Miodowska, Patrycja Bojdo, Bartosz Gąsiorkiewicz, Paulina Koczurkiewicz-Adamczyk, Kamil Piska, Elżbieta Pękala

**Affiliations:** Department of Pharmaceutical Biochemistry, Faculty of Pharmacy, Jagiellonian University Medical College, Medyczna 9, 30-688 Kraków, Poland

**Keywords:** Aclarubicin, Aclacinomycin A, Anthracycline, Antibiotic, Antineoplastic drug, Antitumor therapy

## Abstract

Aclarubicin (aclacinomycin A) is one of the anthracycline antineoplastic antibiotics with a multifaceted mechanism of antitumor activity. As a second-generation drug, it offers several advantages compared to standard anthracycline drugs such as doxorubicin or daunorubicin, which could position it as a potential blockbuster drug in antitumor therapy. Key mechanisms of action for aclarubicin include the inhibition of both types of topoisomerases, suppression of tumor invasion processes, generation of reactive oxygen species, inhibition of chymotrypsin-like activity, influence on cisplatin degradation, and inhibition of angiogenesis. Therefore, aclarubicin appears to be an ideal candidate for antitumor therapy. However, despite initial interest in its clinical applications, only a limited number of high-quality trials have been conducted thus far. Aclarubicin has primarily been evaluated as an induction therapy in acute myeloid and lymphoblastic leukemia. Studies have indicated that aclarubicin may hold significant promise for combination therapies with other anticancer drugs, although further research is needed to confirm its potential. This paper provides an in-depth exploration of aclarubicin’s diverse mechanisms of action, its pharmacokinetics, potential toxicity, and the clinical trials in which it has been investigated.

## Introduction

Aclarubicin (ACR), also known as aclacinomycin A, was first isolated from *Streptomyces galilaeus* culture by T. Oki et al. in 1975 [1]. The microorganism was found in a soil sample collected at Kamiosaki, Japan. As it turned out, the microorganisms produced an antibiotic complex called aclacinomycin, which was then extracted from the cell culture and separated on silica gel. The obtained compounds were examined by NMR and mass spectroscopy. One of them was ACR. Scientists observed ACR ability to inhibit leukemia in mice, which was the impulse to do further research.

**Fig. 1:**
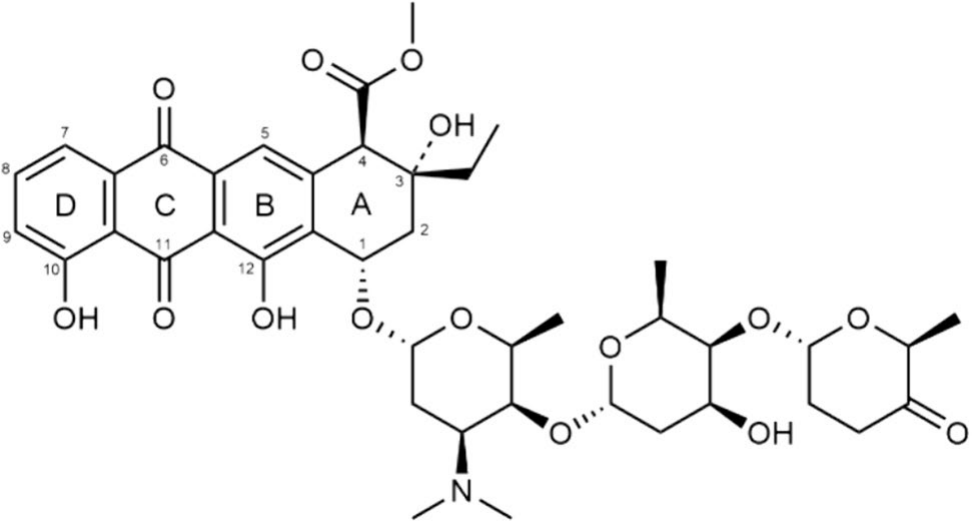
Structure of aclarubicin

ACR is a second generation anthracycline antineoplastic antibiotic with multidirectional mechanism of antitumor and antiproliferative action. The most important of these is inhibition of topoisomerase II binding to DNA. Moreover, ACR interacts with topoisomerase I in a dose depending manner. ACR also induces the formation of free radicals, cell apoptosis and inhibits angiogenesis, has antimetastatic activity and inhibits the activity of chymotrypsin and the 20S proteasome. Furthermore, it can affect the regulation of erythroid gene expression. One of its main advantages over other anthracyclines, such as doxorubicin (DOX) or daunorubicin (DNR), is its low cardiotoxicity [2]. In terms of chemical structure, ACR, like other anthracycline antibiotics, is a glycoside composed of tetracyclic aglycone with a quinone ring. The aglycone moiety, aklavinone, is linked at the C-1 position of ring A to a trisaccharide which contains three deoxyhexoses: L-rhodosamine, 2-deoxy-L-fucose and L-cinerulose A. This is a feature that distinguishes ACR from doxorubicin or daunorubicin, and other anthracyclines, since the latters generally contain only one aminosaccharide group (Fig. 1).

The aim of this work is to review current literature on ACR, with particular emphasis on the multidirectional mechanism of antineoplastic action, pharmacokinetics, toxicity and recently conducted clinical trials.

## Molecular mechanism of action of aclarubicin

### Interfering with DNA transcription and replication

Anthracyclines are well known for their capacity to bind to DNA. This noncovalent binding process of placing the aglycone of the antibiotic between adjacent base pairs of DNA double helix, called intercalation, results in DNA structure change [1]. Intercalation inhibits cell division and growth by blocking the process of DNA replication. This effect is prominent for quickly proliferating cells, such as cancer [3]. Moreover, ACR influences the activity of both topoisomerases. While doxorubicin and daunorubicin are only able to act on topoisomerase II [4], ACR has a dual mechanism of action, acting as a topoisomerase II catalytic inhibitor as well as a topoisomerase I poison [5]. During cell replication process there is a build-up of tension in the DNA. Topoisomerases are enzymes responsible for altering the topology of the DNA through binding and cleaving the strands of the double helix and later rearranging and re-ligating them in a new position [6]. Anthracycline antibiotics work on topoisomerase II in at least two different mechanisms. They may act by stimulating the formation of DNA-topoisomerase II cleavable complex or inhibing DNA cleavage by preventing association of the enzyme and DNA. Studies have shown that ACR belongs to the latter group of drugs [4]. While action of ACR against topoisomerase I is similar to camptothecin. The drug stabilizes topoisomerase I—DNA cleavage complex which causes damage of the DNA structure. In this mechanism, topoisomerase acts as a ‘poison”—it isn't the lack of enzyme activity that causes the destruction of the genetic material but inhibition of enzyme is a specific point of catalytic cycle, after cleave of DNA strand [6].

What is more, anthracyclines present the ability to induce histone eviction from the DNA, which enhances the generation of DNA breaks. Although this mechanism was not observed when the chromatin was fully condensed. While daunorubicin mostly affected active gene bodies, for ACR, the targeted genomic regions were marked by histone modification H3K27me3, hence recognized as facultative heterochromatin, containing repressed as well as silent genes. The additional sugar moieties of ACR may be responsible for its ability to dissociate nucleosomes from more condensed chromatin structures. Targeting relatively silent or less active regions, such as poised promoters, might be useful in treating tumors with that particular epigenetic state [7].

### Antimetastatic potential

Studies have shown that ACR possesses the ability to inhibit cancer cell migration in non-cytotoxic concentrations. Along with decreased cell motility, changes in the cell shape were observed. Cells treated with ACR exhibited very discrete leading lamellae, weaker actin folds distributed along the membrane, vinculin presence only at the cell periphery, loss of polarity and a presence of discontinuous membrane ruffles. This together might be the reason for the impairment of cells migration. The antimetastatic effect has also been achieved by slight increase of cell adhesion. Additionally, the treatment with ACR led to a decrease of cellular distribution and affinity state of β1 integrins—a major class of cell surface receptors which are important for the cell-extracellular matrix interaction and cell migration. The components of integrin signaling, FAK and Src kinase, were also strongly affected by the ACR treatment, with higher phosphorylation level of FAK leading to phosphorylation-dephosphorylation imbalance and suppressed cell motility. These mechanisms together suggest that ACR may affect cell membrane functions and, in addition, suppress the tumor invasion process [8].

### Generation of reactive oxygen species

**Fig. 2:**
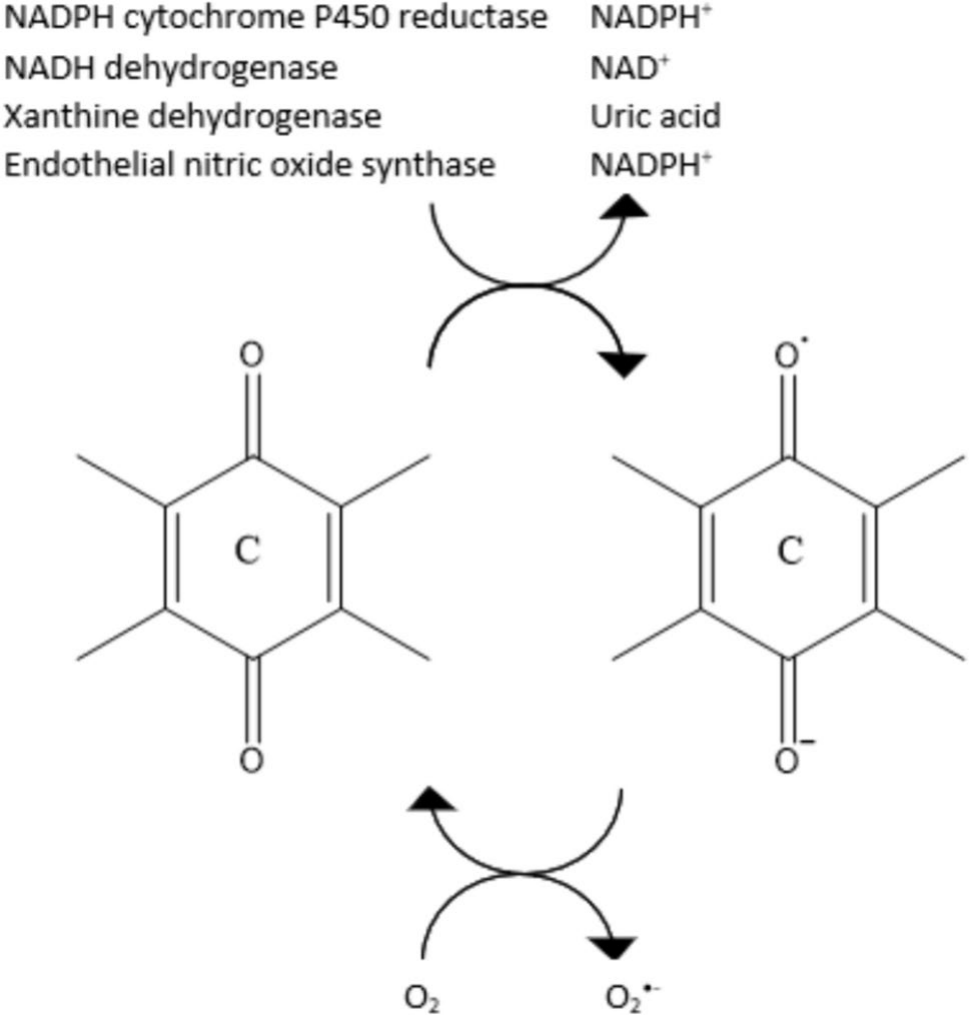
One-electron reduction of anthracycline antibiotics (based on [10] modified)

The generation of reactive oxygen species (ROS) is considered to be one of the mechanisms of ACR cytotoxicity. Mitochondrial Respiratory Complex I is responsible for the reduction of anthracyclines to their semichinone derivatives [9] (Fig. 2).

Those forms in presence of transition metals and molecular oxygen cause oxidative stress and cellular damage by inducing the generation of ROS such as H_2_O_2_ or O_2_^•−^. Until now, iron was considered as an element essential for oxidative DNA damage [11], but recent studies proved that copper has apoptotic activity as well [12]. The production of ROS can lead to the DNA-damage alongside topoisomerase inhibition and DNA intercalation [13]. Furthermore, the metabolism of ACR and ROS production cause the loss of mitochondrial membrane potential, what might potentiate the cytotoxic effect. Compared with doxorubicin, ACR has the ability to generate ROS in lower concentration (IC_50_ = 0.274–0.621 for ACR vs. IC_50_ = 2.842–5.321 for DOX) [14]. ACR can also induce NOS expression and NO release, which may contribute to cardiovascular dysfunction, although not as severe as with other anthracycline antibiotics. Moreover, the activity of NOS also causes a one-electron reduction of anthracyclines, contributing to oxidative stress [15].

### Induction of cell death

ACR, similarly to other anthracycline antibiotics, has been observed to induce biochemical changes leading to programmed cell death, promoting apoptosis with the cell membrane remaining intact [16]. These conclusions have been reached by detecting morphological changes in the tumor cells affected by ACR (concentrations up to 500 nM, incubation time 3–24 h), such as cell shrinkage, chromatin condensation, externalization of phosphatidylserine, DNA fragmentation and formation of apoptotic bodies which are postulated as central criteria of programmed cell death [16, 17]. However, it has been observed that the anti-tumor activity of ACR can lead to either apoptosis or necrosis of the cancerous cells, depending on the kind of cell line, the capability of cells to accumulate the drug, the incubation time and the dose of the drug. According to a study on A549, HepG2, and MCF-7 cell lines, while apoptosis is the major mode of cell death during the first 48 h of the treatment, it can be followed by secondary necrosis that has been detected after longer incubation (72–96 h), with ACR doses corresponding to the IC_50_ values for each cell line (0.27 µM, 0.32 µM and 0.62 µM respectively). The higher ability to accumulate the drug correlated with a higher level of apoptosis [17].

Another study, conducted on human acute lymphoblastic leukemia Jurkat cells, showed that ACR sensitizes cells to TRAIL-induced apoptosis. The reason for this action is the ACR-induced up-regulation of mRNA and protein expression of TRAIL receptor, as well as induction of caspase-8, Fas, and receptor-interacting protein. The action on TRAIL receptor promoter was independent of p53, as opposed to the similar effect achieved during treatment with doxorubicin [18].

### Inhibition of chymotrypsin-like activity of the 20S proteasome

The potential anti-tumor activity of ACR is also related to its influence on the process of ubiquitin-dependent protein degradation. This process is carried out by the 20S proteasomes and the 26S proteasomes (whose catalytic center consists of the 20S proteasome). Proteasomes are macromolecular protein complexes directly involved in the breakdown of most polyubiquitinated proteins and polypeptides in eukaryotic cells. They are responsible for the degradation of nuclear oncoproteins and cyclins, which play a key role in regulating the cell cycle. Among many active sites of the proteasome, chymotrypsin-like, trypsin-like, and caspase-like sites can be distinguished [19, 20].

The mechanism of action of ACR differs from that of another anticancer drug, which affects the process of ubiquitin-dependent protein degradation—cisplatin. Cisplatin acts directly on ubiquitin, influences the process of attaching ubiquitin to proteins and polypeptides. In vitro studies on rabbit reticulocyte lysates have shown that ACR acts by inhibiting the activity of the catalytic centers of the 20S proteasomes, without affecting the ubiquitination process (IC_50_ = 52 µM) [21]. Moreover, ACR at a concentration of 50 µM was shown to significantly inhibit only the chymotrypsin-like activity of the 20S proteasome, without affecting the trypsin-like and caspase-like activity [20, 22]. The effectiveness of this mode of activity in producing an overall anticancer effect is uncertain, primarily because it necessitates relatively high concentrations of ACR for activation.

### Inhibition of angiogenesis

Hypoxia-inducible factor- 1 (HIF-1) is a transcription factor responding in cells due to the low oxygen concentration. Under hypoxic conditions, HIF-1 has the ability to translocate to the nucleus, where it initiates the expression of specific genes involved in the tumor progression process such as VEGF [23]. In turn, VEGF is one of the most significant factors that stimulate angiogenesis and neovascularisation, and without its crucial role, this process cannot occur. ACR can inhibit the activity of HIF-1 and consequently inhibits the hypoxic induction of VEGF. Yamazaki et al. conducted an experiment that proved the above statement. They transfected a mammalian cell line with a luciferase reporter gene construct which contained a few copies of HIF-1 binding site. The activity of luciferase under hypoxic conditions was inhibited by ACR (IC_50_ = 0.021 mg/ml *i.e.* 25,89 µM) but neither doxorubicin nor daunorubicin showed that effect. In addition, ACR reduced VEGF protein expression in a dose-dependent manner while doxorubicin and daunorubicin showed no such effect. Unlike the above drugs, ACR affects topoisomerase I, which, according to the authors of the study, may have consequences in inhibiting angiogenesis by ACR [24].

### Regulation of erythroid gene expression

**Fig. 3:**
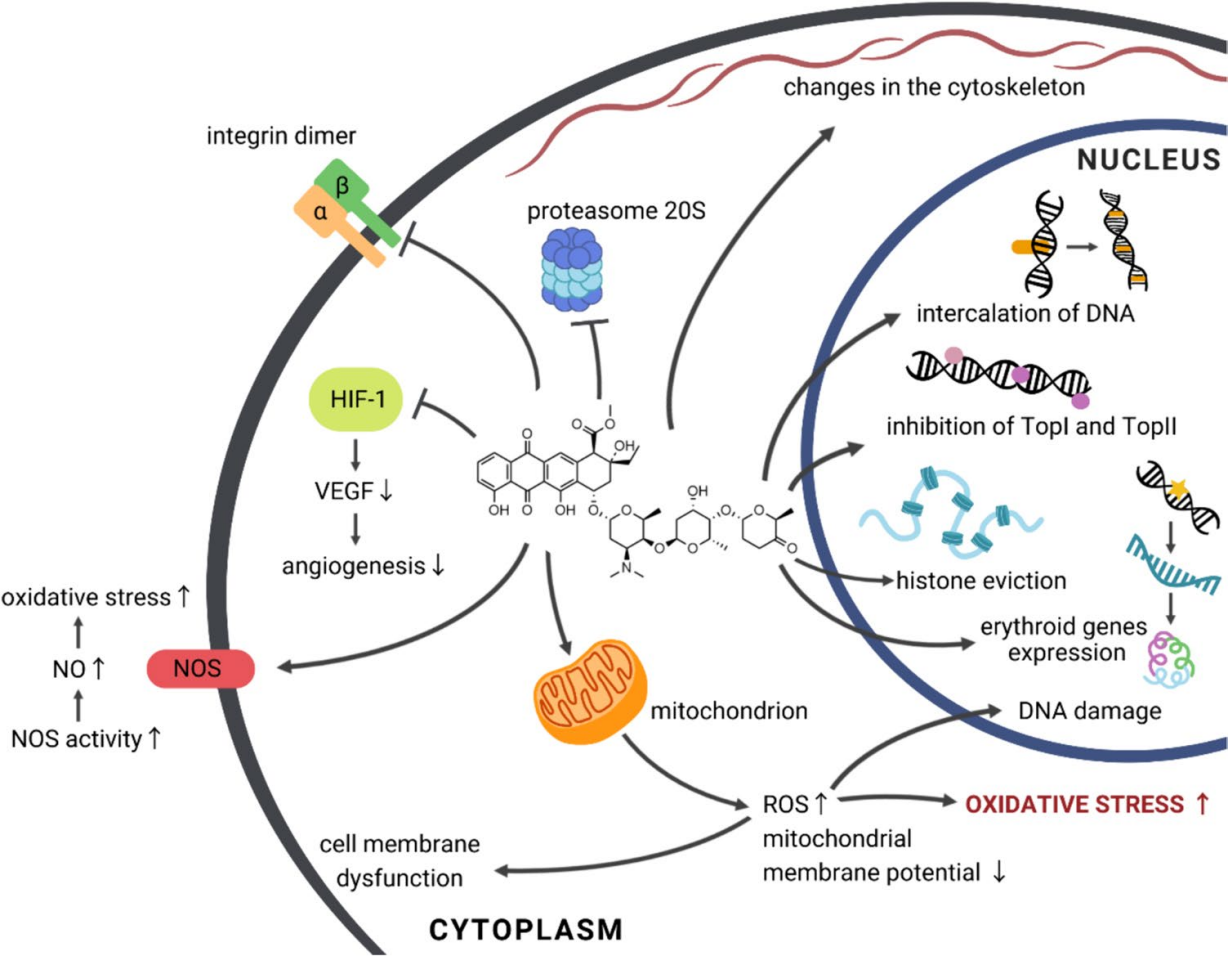
Molecular pathways initiated by aclarubicin

The concept that leukemic cells are immature due to differentiation blocks and cannot thus control their growth created a therapeutic alternative for the patients [25, 26]. Instead of the destruction of cells, the agents could resume their maturation process. Furthermore, differentiation agents could show reduced toxicity. ACR, as well as some other anthracyclines, in sub-toxic concentration, are considered differentiation inducing drugs. In vitro studies have proven ACR can affect the regulation of erythroid gene expression in leukemic and solid tumor cells (e.g., neuroblastoma, melanoma) [27, 28]. After administration of low doses of ACR to patients with Acute Myelomonocytic or Myeloblastic Leukemia, the rise in the appearance of mature cells was noted. The concentrations causing cell differentiation ranged widely from 30–50 nM [28] to 250–350 nM [27], depending on the cell line used in the experiment. On the other hand, these reports are based on only a single human cases [29, 30]. Patients with terminal acute myeloblastic leukemia were administered 7 to 20 mg/daily of ACR. After the course, an increase in the number of mature neutrophils was noted along with a decrease of leukemia cells. The exact mechanism is still poorly understood; however, a few conclusions have been drawn. ACR acts mainly by regulating the transcription. It involves inducing the expression of erythropoietin genes, such as gamma-globin, PGBD, erythropoietin receptor (EpoR), and overexpression of erythroid transcription factors, such as GATA-1 [31] (Fig. 3).

### Chemosensitization and radiosensitization

One of the investigated therapeutic possibilities of ACR is its support in subclinical doses during doxorubicin therapy. ACR is postulated to sensitize cells to the action of DOX. In addition, administering two drugs simultaneously reduces the risk of resistance. Cross-resistance of the anthracyclines: DOX and DNR were observed on the leukemic cell line, but this effect did not apply to ACR. ACR may act as a multi-drug resistance modulator. It has been suggested that ACR may be an inhibitor of proteins involved in drug resistance. The phenomenon of cross-resistance occurs in many cancers, hence great hopes are placed in combination therapy with drug resistance modulators [32]. Yueh-Lun Lee et al. in their research focused on the possibility of sensitizing K562 myeloid leukemia cells to imatinib (at the concentration of 200 nM), by 10 nM ACR co-treatment. Imatinib is a specific Bcr-Abl tyrosine kinase inhibitor and is used as first-line treatment for short-term chronic myelogenous leukemia (CML). The difficulty in treating CML is the resistance of the tumor progenitor cells. ACR induced erythroid differentiation via the mitogen-activated kinase38 (MAPK) pathway. The inhibition of erythroid differentiation by p38MAPK inhibitor, p38MAPK mutation, or p38MAPK knockdown, reduced the effectiveness of ACR/imitanib treatments influence on growth inhibition and apoptosis. These results suggest that ACR-induced differentiated K562 cells are more sensitive to imatinib. Moreover, such a scheme led to inhibition of their growth and induction of apoptosis, down-regulation of Bcr-Abl, Mcl-1, and Bcl-xL, as well as activation of caspase-3 [33].

ACR also shows synergism in combination with arsenic trioxide. Arsenic trioxide is a substance used to treat acute promyelocytic leukemia. This is related to the regulation of apoptosis-related proteins, including Bcl-2 down-regulation and caspases activation. It is also currently being tested for the treatment of other cancers. As_2_O_3_ therapy is limited by its side effects, including gastrointestinal and cardiac toxicity. A study by Yongbin Ye et al. showed that administration of a low dose of As_2_O_3_ and ACR in a ratio of 40: 1 (e.g. 0.4 mM to 10 nM) gave positive results. This treatment regimen reduced Bcl-2, c-IAP and XIAP, and significantly activated caspase-3 and SMAC. These results indicate activation of apoptotic pathways and arrest of the cell cycle, which gives a chance to improve the effectiveness of the therapy based on the synergy of As2O3 and ACR [34].

Interesting results were obtained in the context of combining ACR with radiotherapy. The results show that ACR reduces the expression of glycoproteins in the plasma membrane, including the epidermal growth factor receptor (EGFR) and Met. Moreover, ACR lowers the level of RTKs (receptor tyrosine kinases) associated with tumor growth, angiogenesis, and increased risk of metastasis. Following this path, studies have been carried out confirming that ACR sensitizes cancer cells to radiation, the survival of which depends on RTK signaling. The results confirm that 2-h pretreatment with 500 nM of ACR lowers the level of RTK protein and increases the sensitivity to radiation [35].

## Resistance

The results of in vitro studies carried out on various cell lines (including hepatoma, SCCL and P388 leukemia cells) indicate that the resistance of cancer cells to ACR is lower than that to traditional anthracyclines, such as doxorubicin, daunorubicin or epirubicin. It has been suggested that the two main causes of this phenomenon are related to the increased expression of P-glycoprotein in neoplastic cells and the differences in the mechanism of action between ACR and, DNR and EPI.

To ensure an adequate biological effect, both in in vitro studies and after administration of the above-mentioned anthracyclines to patients, an important aspect is to maintain the optimal concentration of the drug in the tumor cells for a specified time. It has been proven that the resistance of neoplastic cells to these anthracyclines is largely associated with a decrease in the concentration of the drug in the cells, of which the main reason is the increase in the expression of P-glycoprotein in these cells. However, because ACR is a weak substrate of P-gp, high levels of ACR were observed in cells with increased expression of this glycoprotein, while concentrations of other anthracyclines were significantly reduced [36]. The different mechanisms of drug accumulation in cells may also explain the absence or weak cross-resistance to ACR in cells resistant to DOX or DNR [37, 38]. The decreased resistance to ACR is also related to the fact that, unlike the other discussed anthracyclines, ACR activity is not dependent on the intracellular level of topoisomerase II, the amount of which increases in the S phase of the cell cycle. For this reason, the ACR effect is noticeable even with a small number of S-phase cells [39].

## Pharmacokinetics

ACR is an intravenous, rapidly metabolized drug with half-life of 13.3 h. The metabolism of ACR takes place in the liver and leads to the formation of inactive aglycons and cytotoxic glycosides, with a half-life of up to 25 h. ACR is mainly excreted in the bile, but about 6% is also excreted in the urine. The volume of distribution determined in preclinical studies in mice is 39.1 l/kg; the drug accumulates in the tissues and is then released from the tissues into the blood [40, 41]. During preliminary studies in humans, it was possible to establish that ACR has a two-compartment drug distribution model, like other anthracyclines. ACR was characterized by volume of distribution of 2073 L/m^2^ and a the total body clearance of 150 L/m^2^/h [41].

## Clinical trials

Despite the initial interest regarding clinical applications of ACR, few high-quality trials were conducted to evaluate the drug’s efficacy in recent years. The main rationales behind ACR use in oncology are: (i) lack of cross-resistance with other anthracyclines and (ii) lower cardiotoxicity which were postulated after in vitro studies. In theory, these make ACR the perfect choice for therapy of neoplasms that were resistant to previous regimens consisting of anthracyclines or relapsed cases.

Treatment regimens based on ACR alone or in combination with other drugs were mainly evaluated as induction therapy in acute myeloid and lymphoblastic leukemia (AML and ALL). The main outcomes of those studies, regarding the clinical efficacy of tested regimens, are summarised in Table 1, while details on the regimens of the discussed therapies are presented in Table 2.

**Table 1:** Studies on the use of ACR for systemic anti-neoplastic therapy

Type of study	No. of patients enrolled	Disease	Intervention	Main outcomes	Reference
Case series	22	AML – 9 pts (resistant to previous therapy) Leukemia lymphosarcoma – 8 pts	Aclarubicin (10- 20 mg/m^2^ daily; total dose varied from 42 to 600 mg/m^2^)	4 pts achieved CR or PR 4 pts achieved CR or PR Significant haematologic toxicity, low level of cardiac toxicity and no alopecia recorded	[42]
Case series	46	refractory and/or relapsed AML	HAA induction regimen	CR 76,1% after single course Estimated 3-year OS 42% Estimated RFS after CR at 3 years 49% Acceptable toxicity including no cardiac toxicity	[43]
Case series	36	refractory or relapsed AML with translocation (8;21)	CAG nduction regimen	Overall CR 75% Median CR duration 12 months 2-year LFS 25% No significant treatment-related adverse events observed, frequent myelosuppression, nonhematologic toxicities were comparatively mild	[44]

Case series	37	Refractory or relapsed AML	Aclarubicin: 60 mg/m^2^ /day and etoposide 100 mg/m^2^ /day each given for 5 days in induction therapy	CR 24% PR 16% 22% early deaths (within 6 weeks) OS 3.2 months DFS 3.2 months Acceptable toxicity: hematologic (neutropenia) and non-hematologic side-effects (stomatitis, infections, nausea/vomiting, diarrhea); only mild cardiac toxicity reported (no serious events recorded; mild heart arrhythmia in 17% and mild heart function defect in 5% of pts)	[45]
Case series	10 1	Refractory or relapsed AML CML in blast crisis	ACR + etoposide induction regimen	CR 36% Median duration of CR 224 days 5-year OS 27% vs 20% (not significant) 10-year OS 24% vs 16% (not significant) Early deaths 5/11 Cardiac toxicity: mild (7/19 cycles applied), severe (1/19 cycles applied) Haematological toxicity (neutropenia, thrombocytopenia, five patients died in aplasia) Non-haematological toxicity (infection, hepatic toxicity, diarrhoea, mucositis, nausea/vomiting)	[46]
RT	174	De novo AML in pts under 65	ACR + ara-C vs DNR + ara-C induction regimens	5-year OS 27% vs 20% (not significant) 10-year OS 24% vs 16% (not significant)	[47]

Case series	32	AML in elderly pts (60–76 yo)	AVA regimen in induction and consolidation treatment	CR 53%, PR 6%, Median DFS 12 months, Median survival 16.6 months, Overall treatment related deaths 16% Significant, but acceptable toxicity with no significant cardiac toxicity reported, non- hematologic including stomatitis, nausea, vomiting, and diarrhoea, skin reactions	[48]
RT	90	Elderly patients (80–89 yo) with newly diagnosed AML	TAD vs TAA induction regimen	CR: 51% vs 47% (no difference) Median durations of first remission 11.6 vs 10.7 month. Additionally: Early deaths (within 30 days) 7/43 vs 17/47 pts No difference in cause-specific long-term survival (at > = 10 years of follow-up) Similar toxicity profile in both regimens (nausea, oral mucositis, diarrhoea, and alopecia);cardiac toxicity cannot be evaluated	[49]
RT	360	Previously untreated AML in pts < 65yo	BH-AC + DMP vs BH-AC + AMP regimens in induction and consolidation therapy	CR 63,7% vs 53,9% (not significant) Median DFS in pts achieving CR: 15,4 months vs 14,1 months; 7-year DFS 21,1% vs 27,7% (not significant); Median survival 15,8 months vs 9,5 months; 7-year survival rate 19,3% vs 20,2%^b^ (Comparable effects). Diarrhea, ileus, pneumonia, and renal failure were more frequent with BH-ACo-AMP than with BH-ACo- DMR	[50]

Case series	68	Previously untreated AML in elderly pts (60–70 yo)	CAG regimen as induction and maintenance therapy (in those achieving CR)	CR 49% Early mortality (8 weeks) 22% In pts who achieved CR: median duration of DFS: 10 months Median OS: 9 months 4-year OS: 13.8% 4-year DFS: 9.4%	[52]
Case series	9 receiving HAA	MDS-RAEB and RAEB-T	HAA induction regimen	CR after first course: 67% estimated 3-year OS: 76% “well-tolerated”	[53]
Case series	48	De novo AML	HAA regimen in induction and consolidation therapy	CR 83% 3-year OS 53% Early death 4% Acute heart arrythmia and transient reduction in LVEF in one pts Acceptable toxixity compared with other regimens, the most common non- hematological toxicity was infection (pneumonia, acute bronchitis and tracheitis)	[54]

RT	609	De novo AML	HAA vs HAD vs DA induction regimens	CR: 73% vs 67% vs 61%^a^ OS at 3-years: 44,5% vs 43,5% vs 42,7%^a^ Event-free survival at 3-years: 35,4% vs 32,7% vs 23,1%^a^ ^a^statistical analysis provided for HAA vs DA – HAA superior regarding CR and event-free survival Similar non-haematological and haematological toxic effects among groups except for early deaths (within 30 days) – 5,8% vs 6,6% vs 1%	[55]
RT	98	AML (patients of low and intermediaterisk groups with unsuccessful first induction treatment attempt)	HCAG vs FLAG re-induction regimens	Similar efficacy (CR 51,1% vs 54,9%—no difference; OOR 76,6% vs 82,4%—no difference; median PFS 29.8 months vs 30.8 months) Lower haematologic and nonhaematologic toxicity in HCAG	[56]
RT	39	Previously untreated MDS	ACR (10 mg/m^2^xday in 2 courses of 10 days) vs ara-C (3 mg/m 2, twice daily for 21 days)	No significant difference in the therapeutic effects and survival between these two groups of patients	[57]

Case series	40	Relapsed AML	ACR 100 mg/m^2^xday (day 1–3) repeated on days 14–16 if no marrow hypoplasia present, induction and consolidation therapy	CR 27,5% No pt presented decreased LVEF or clinical cardiac symptoms Minimal alopecia Myelosupression as major toxicity effect; diarrhea, mucosistis, nausea/vomitting	[58]
Case series	38	Refractory AML	ACR: 10 to 30 mg/m^2^ i.v. bolus until the maximum total dose of 300 mg/m^2^ per course was reached or until unacceptable toxicity (13 pts) 10-day courses of ACM at the daily dose of 15 mg/m2 i.v. bolus with 10-day intervals between courses (25 pts)	Overall CR 35% CR 15% CR 44% CR in patients (17) refractory to previous treatment with DNR/ADM 35% Acceptable toxicity, mucositis, diarrhea, vomiting and infection No alopecia observed; ECG changes or arrythmias in 4 pts	[59]
Case series	44	Possibly refractory acute non- lymphocytic leukemia previously treated with DNR and cytarabine	80 mg/m^2^ of ACR iv daily for 3 days, followed by 80 mg/m^2^ iv daily for 2 days in patients not obtaining a complete remission (CR) after 2–4 weeks monthly maintenance chemotherapy with ACR and cytarabine	CR 18% PR 14% Duration of CR 10–58 weeks No case of chronic cardiotoxicity recorded; acute cardiotoxicity suspected in 7 pts Mucositis and alopecia were uncommon, frequent nausea and vomiting, diarrhea	[60]

RT	174	De novo AML in pts < 65 yo	ACR (75 mg/m^2^ daily for 3 days) + ara-C (100 mg/m2 per day for 7 days) vs DNR (45 mg/m2/day for 3 days) + ara-C (100 mg/m2 per day for 7 days)	CR 66% vs 50% (significant) 4 year DFS in those achieving CR 37% vs 33% (not significant) 4-year OS 29% vs 20% (not significant) Identical hematological toxicity	[61]
Case series	15	MDS and AML that progressed from MDS	ACR 3–14 mg/m2xday, two courses of 7- 10 days	CR in 3 pts with MDS and 1 pt with AML Myelosuppression caused by low-dose ACR was milder than that caused by low-dose Ara-C	[62]
Case series	38	ALL with early bone marrow relapse (all pts previously received heavy treatment including anthracyclines) in children	Prednisolone + AVA	CR 57,9% Early deaths 15,8% One cardiac failure recorded 5 patients died from hemorrhages or infectious complications. The main side effects were fever, gastrointestinal problems, stomatitis, and severe bone marrow aplasia	[63]

Case series	14	Multiple myeloma	ACR i.v	9/15 patients achieved > 25% reduction of M- protein (including 2 patients with > 50% reduction) cardiac toxicity was not detected and epilation was mild	[64]

*RT* randomised trial, *AML* acute myeloid leukemia, *CR* complete remission, *PR* partial remission, *OOR* objective response rate, *ORR* overall response rate, *PFS* progression free survival, *RFS* relapse free survival, *LFS* leukemia free survival, *DFS* disease free survival, *RAEB*—refractory anemia with excess of blasts, *RAEB-T* refractory anemia with excess of blasts in transformation, *DNR* daunorubicin, *ACR* aclarubicin, *ADM* Adriamycin, *Ara-C* cytosine arabinoside

^a^analysis provided for HAA vs DA – HAA superior regarding CR and event-free survival

^b^difference between survival curves statistically significant in generalised Wilcoxon test and statistically nonsignificant in log-rank test

**Table 2:** Treatment regimens with ACR in research studies

Therapy abbreviation	Therapy regimen			Reference
	Drug	Dose	Dosing period	
HCAG	Cytarabine	10 mg/m^2^/12 h	On 1–14 day	[56]
	Aclarubicin	7 mg/m^2^/day	On 1–8 day	
	G-CSF	200 μg/m^2^/day	On 1–14 day	
	HHT	1.5 mg/m^2^/day	On 1–8 day	
FLAG	Fludarabine	30 mg/m^2^/day	On 1–5 day	[56]
	Cytarabine	2 g/m^2^/day	On 1–5 day	
	G-CSF	300 μg/day	On 1–5 day	
D-CAG	Decitabine	–	On 1–5 day	[51]
	G-CSF	–	On 1–9 day	
	Cytarabine	–	On 3–9 day	
	Aclarubicin	–	On 3–6 day	
HAA	HTT	2 mg/m^2^/day	On 1–7 day	[55]
	Cytarabine	100 mg/m^2^/day	On 1–7 day	
	Aclarubicin	20 mg/day	On 1–7 day	
HAD	HHT	2 mg/m^2^/day	On 1–7 day	[55]
	Cytarabine	100 mg/m^2^/day	On 1–7 day	
	Daunorubicin	40 mg/m^2^/day	On 1–3 day	
DA	Daunorubic	40–45 mg/m^2^/day	On 1–3 day	[55]
	Cytarabine	100 mg/m^2^/day	On 1–7 day	
HAA	HHT	4 mg/m^2^/day	On 1–3 day	[43]
	Cytarabine	150 mg/m^2^/day	On 1–7 day	
	Aclarubicin	12 mg/m^2^/day	On 1–7 day	
CAG	Ara-C	10 mg/m^2^/12 h	On 1–14 day	[44]
	Aclarubicin	6 mg/m^2^/day	On 1–8 day	
	G-CSF^a^	200 g/m^2^/day	On 1–8 day	
HAA	HHT	2 mg/m^2^/12 h	On 1–3 day	[53]
	Ara-C	75 mg/m^2^/12 h	On 1–7 day	
	Aclarubicin	12 mg/m^2^/day	On 1–7 day	
IA	Idarubicin	12 mg/m^2^/day	On 1–3 day	[53]
	Ara-C	75 mg/m^2^/12 h	On 1–7 day	
CAG-LD	Ara-C	10 mg/m^2^/12 h	On 1–14 day	[52]
	Aclarubicin	14 mg/m^2^/day	On 1–4 day	
	G-CSF^b^	200 g/m^2^/day	On 1–14 day	
TAD	Thioguanine	100 mg/m^2^/12 h	On 1–7 day	[49]
	Ara-C	100 mg/m^2^/12 h	On 1–7 day	
	Daunorubicin	60 mg/m^2^/day	On 5–7 day	
TAA	Thioguanine	100 mg/m^2^/12 h	On 1–7 day	[49]
	Ara-C	100 mg/m^2^/12 h	On 1–7 day	
	Aclarubicin	80 mg/m^2^/day	On 5–7 day	
AVA	Aclarubicin	25 mg/m^2^/day	On 1–4 day	[48]
	Etoposide	100 mg/m^2^/day	On 1–3 day	
	Ara-C	100 mg/m^2^/day	On 1–3 day	
	Ara-C	100 mg/m^2^/12 g	On 4–7 day	
	Alcarubicin	75 mg/m^2^/day	On 1–3 day	[47]
	Daunorubicin	45 mg/m^2^/day	On 1–3 day	
	Ara-C	100 mg/m^2^/day	On 1–7 day	
	Aclarubicin	60 mg/m^2^/day	On 1–5 day	[46]
	Etoposide	100 mg/m^2^/day	On 1–5 day	
BH-AC + DMP/AMP	BH-AC	170 mg/m^2^/day	On 1–10/14 day	[50]
	6MP	70 mg/m^2^/day	On 1–10/14 day	
	PSL	20 mg/m^2^/day	On 1–10/14 day	
	Daunorubicin	25 mg/m^2^/12 h	On 1–2 day	
	Aclarubicin	14 mg/m^2^/12 h	On 1–10/14 day	
Prednisone + AVA	Prednisone	100 mg/m^2^/day	On 1–7 day	[63]
	Ara-C	100 mg/m^2^/12 h	On 1–7 day	
	Aclarubicin	25 mg/m^2^/day	On 1–5 day	
	Etoposide	150 mg/m^2^/day	On 5–7 day	
HAA	Homoharritonine	4 mg/m^2^/day	On 1–3 day	[54]
	Cytarabine	150 mg/m^2^/day	On 1–7 day	
	Aclarubicin	12 mg/m^2^/day	On 1–7 day	

*HHT* homoharringtonine, *G-CSF* granulocyte colony-stimulating factor, *BH-AC* behenoyl cytosine arabinoside, *6MP*—6-mercaptopurine, *PSL* prednisolone, *DMP* daunorubicin, 6-mercaptopurine and prednisolone, *AMP*—aclarubicin, 6-mercaptopurine and prednisolone, *DNR* daunorubicin, *ACR* aclarubicin, *Ara-C* cytosine arabinoside

^a^unless patient’s white blood cell (WBC) count was ≥ 20 × 109/L

^b^withdrawn when the leukocyte count was > 10 × 109/L

In several published case series papers ACR was found to be mildly effective in remission induction in patients with refractory or relapsed AML. Despite low CR rates (between 18 and 37%), ACR seems to be a promising candidate for combined therapy regimens, especially considering the characteristic of the evaluated group. Although a significant proportion of enrolled patients were primarily resistant to or relapsed after previous therapy with other anthracyclines, they responded to ACR, which may confirm the lack of cross-resistance between the drugs [42–46]. However, those results should be interpreted with caution due to the small sample size and lack of control groups. In addition, Mathé et al. provided preliminary data regarding the use of ACR in the treatment of refractory ALL and lymphosarcoma [42].

Furthermore, two trials compared ACR in combination with cytosine arabinoside (ara-C) and daunorubicin puls ara-C in patients with newly diagnosed AML. No significant difference was found in CR rate or long-term survival between groups [47].

ACR in combination with etoposide was found to induce remission in some patients with refractory or relapsed AML and was proposed as an alternative treatment for patients with an allergic reaction to ara-C [45, 46]. Moreover, AVA protocol, consisting of ACR, ara-C, and etoposide was proposed as a treatment option for remission induction and consolidation in elderly patients with AML [48]. Fengler et al., on the other hand, evaluated the efficacy of AVA with prednisone in heavily pre-treated children with ALL bone marrow relapse [58].

Ӧberg et al. compared the response to induction regimens consisting of thioguanine, ara-C, and either ACR or DNR in elderly patients with newly diagnosed AML and found no statistically significant difference in CR or long-term cause specific mortality [49].

Furthermore, a randomized trial by Nagura et al. found no statistically significant difference in CR and 7-year DFS between regimens consisting of behenoyl cytosine arabinoside, 6-mercaptopurine, prednisolone and either ACR or DNR used for induction and consolidation in patients under 65 years old with newly diagnosed AML. Although no difference between the two arms was noted in 7-year survival as well, the DNR group seemed to have better survivability in the early years after diagnosis [50].

The successful use of induction and maintenance regimen CAG, comprising ara-C, ACR, and G-CSF (granulocyte colony-stimulating factor), was reported in patients with newly diagnosed or refractory/relapsed AML. Furthermore modified CAG with the addition of decitabine was proposed as induction treatment in elderly AML patients [44, 51, 52].

The efficacy of combination therapy with homoharringtonine (currently known as omacetaxine mepesuccinate—a clinically approved protein synthesis inhibitor targeting ribosomal subunit A), Ara-C, and ACR (HAA regimen) as an option for remission induction and consolidation in patients with de novo or refractory/relapsed AML was suggested by three case series studies [43, 53, 54]. Moreover, the results of a randomised trial conducted by Jin et al., which enrolled patients with de novo AML, proved the superiority of HAA induction regimen over DNR alone [55].

Zhang et al. compared HCAG therapy (CAG + Homoharringtonine) with FLAG (fudarabine, ara-C and G-CSF) as induction regimens in patients with refractory AML and found no statistically significant difference in CR and OOR [56].

Although it is important to note the scarcity of large high-quality studies in the area, the aforementioned trials encourage the use of ACR, especially in AML. The efficacy of ACR alone, however limited, in relapsed cases that were previously treated with other anthracyclines is especially promising. Furthermore, it appears that ACR may be successfully incorporated into combination treatment regimens for better clinical efficacy. Although most of the discussed studies were case series trials, a few randomized trials found no or little difference between widely used AML induction therapy protocols and those containing ACR.

Although cautious optimism may be warranted regarding the potential application of ACR in the treatment of AML or ALL, further high-quality studies are required to precisely establish its clinical efficacy.

Individual papers reported the use of ACR in other hematological diseases—multiple myeloma and myelodysplastic syndrome (MDS) (summarised in Table 1). In the latter case, Harada et al. found no significant differences in therapeutic effects and survival after treatment with either ACR or ara-C [57]. Furthermore, Xu et al. reported HAA regimen to be effective in remission induction for patients with MDS [53].

Similarly, a few studies evaluated the clinical efficacy of ACR in the treatment of solid tumors (Table 3). Generally administered ACR alone was found to have little or no effect in non-small cell lung, small cell lung, breast, pancreatic and gastric cancer, although one study suggested it may have radiosensitizing properties. Conversely, local administration of ACR preparations alone or in combination with other drugs was reported to induce at least some response in this case. This discrepancy may be attributed to pharmacokinetics or cellular uptake of the drug.

**Table 3:** Studies on the use of ACR for local anti-neoplastic therapy

Disease	Intervention	Main outcomes	Reference
Metastatic NSCLC	ACR iv	No effect	[65]
SCLC with previous treatment failure	ACR iv	No effect	[66]
Intracranial alveolar rhabdomyosarcoma	CPT iv + ACR iv	Effective in remission induction	[67]
Lung, stomach and esophagus cancer (advanced or relapsed)	Radiotherapy + ACR iv	ACR may have radio-sensitizing properties	[68]
Metastatic breast cancer	ACR iv	Ineffective	[69]
Metastatic or unresectable pancreatic cancer	ACR iv	Ineffective	[70]
Advanced prostate cancer (relapsed after antiandrogen therapy or hormone-resistant)	ACR iv + CPT iv + CQ iv	May be effective—further study required	[71]
Inoperable, advanced gastric cancer	RT comparing mitoxantrone vs etoposide vs ACR vs spirogermanium	Low response rates and high toxicity of regimens	[72]
Epithelial ovarian cancer after cytoreductive surgery	RT comparing PVB vs CAP regimens in adjuvant therapy	PVB had higher efficacy in remission induction; no statistical difference in long-term (90 months) survival rate (33% vs 16.4%)	[73]
Locally advanced cervical adenocarcinoma	Neoadjuvant therapy with cisplatin, ACR and mitomycin-C (iv or ia) prior to radical surgery	May improve surgical conditions; may improve survival rate	[74]
Advanced gastric cancer with lymph nodes metastasis	Activated carbon adsorbed ACR injected submucosally in the tumour’s vicinity prior to the operation	Improved survival rate compared to surgery alone	[75]
Locally advanced unresectable cervical cancer	CPT + ACR + bleomycin ia or iv	Partial responses achieved in patients receiving ia treatment	[76]
Hepatic metastasis of colorectal cancer	5-FU and lipiodol-ACR in intraportal infusion after curative hepatic resection	Improved survival rate at early postoperative period	[77]
Neoplastic pericardial effusion	ACR intrapericardially	May be highly effective in limiting malignant pericardial effusion	[78]

*RT* randomised trial, *SCLC* small cell lung cancer, *NSCLC* non- small cell lung cancer, *CPT* cisplatin, *ACR* aclarubicin, *CQ* carboquone, *PVB* cisplatin, vinblastine and bleomycin, *CAP* cyclophosphamide, aclarubicin and cisplatin

## Toxicity

Preclinical studies on animal models have proven relatively low toxicity of aclarubicn with maintained antitumor activity (Table 4). In a study conducted on golden hamsters, ACR appeared to cause less apparent changes in the myocardium compared to doxorubicin and daunorubicin. Also, these changes were reversible, degenerative skin changes were not observed. Oki et al. study on beagle dogs indicated weight loss, nausea, and vomiting as adverse effects with 3 months treatment schedule. Myelosuppression also developed and was dose-dependent [79].

Concerning cardiotoxicity, a study on golden hamsters showed that ECG changes after a single intravenous dose were slight to moderate and in multiple administration—slight as well, and reversible after 2 months [80]. Oki et al. study on rabbits have proven these findings. ACR appeared to be more than 10 × less cardiotoxic than doxorubicin as a reference, regarding both acute and chronic cardiotoxicity [79].

**Table 4:** Toxicity of aclarubicin in animal studies

Animal model	Intervention	Main outcomes regarding toxicity	Reference
Golden hamsters	single intravenous administration of 50—75 mg/kg	ECG changes slight to moderate (T wave reduction and occasional arrhythmia, auriculo-ventricular block, T wave)	[80]
	multiple intraperitoneal injections for 16 days; a cumulative dose of 24 mg/kg	reversible R-wave amplitude elevation	[80]
Golden hamsters	multiple intravenous injections for 3 times/week in 1—4 weeks; 6 mg/kg	no significant changes in the myocardium, no degenerative skin changes	[81]
Mice, rats, dogs	daily intraperitoneal or intravenous injection in various dose schedules	weight loss, nausea, vomiting, bone marrow suppression, hepatic impairment	[79]
Golden hamsters	single intravenous administration of 100 mg/kg	transient arrhythmia, reversible ECG alterations	[79]
Rabbits	multiple intravenous injections, 4 and 8 mg/kg/week for 13 weeks	reversible and not significant changes; slight swelling of mitochondria and rare myofibrillar lysis of myocardium	[79]
Hamsters	intravenous, administration of 5 mg/kg	slight ECG changes and modifications of the myocardium, cardiac toxicity lower than doxorubicin	[82]

A general review on the toxicity of aclarubicn in various clinical studies was included in Table 1.

Myelosuppression, especially thrombocytopenia, with often subsequent infections, seems to be the most common effect. Also, nausea, vomiting, and diarrhea are observed [45]. These findings are supported by Yu et al. study in which all patients developed severe granulocytopenia and thrombocytopenia, as well as often gastrointestinal toxicity (nausea, vomiting) [43]. Jianyong et al. have similar observations with rare non-hematological toxicities [51].

The randomized study in acute myeloid leukemia patients noted patients treated with aclarubicn (HCAG regimen) experienced lower toxicity than patients with flutarabine (FLAG regimen) regardless of the grade of hematological toxicity or the presence of non-hematological toxicity (oral cavity toxicity, hepatic dysfunction, renal impairment, pulmonary infection and gastrointestinal disorder) [56]. No alopecia was recorded, or it was uncommon [42, 58].

Aabo et al. suggested that cardiotoxicity of ACR may be higher than it was presented in preclinical studies with patients developing cardiac dysfunction shown by clinical signs and changes in ECG [83]. Other studies observed no significant cardiotoxicity effect with only slight cardiac events (mild heart arrhythmia in mild heart function defect) and no change in left ventricular ejection fraction in post-treatment examination [45, 48, 58]. However, individual patients can develop acute heart arrhythmia or other cardiac complications causing discontinuation of treatment [54, 84]. A possible explanation for the weak cardiotoxic effect of ACR may be due to the relatively low distribution of the drug in the heart [79]. This aspect seems to be a great advantage of this anthracycline, nevertheless, more studies should be conducted, concerning especially the effect on myocardium.

Regarding the maximum dose, the acceptable toxicity limit appears to be 120–150 mg/m^2^ per course. Myelosuppression is considered the main dose-limiting toxicity [42]. Higher doses increase the incidence and intensity of myelosuppression, as well gastrointestinal side effects, such as diarrhea or vomiting. Cardiac events also can be more frequent [59, 60, 83].

## Conclusions

Due to the complexity of its activity, ACR is a relatively poorly understood anthracycline. Apart from inhibition of topoisomerase I and II it was found to affect several other biochemical pathways of cancer cells, leading to anticancer effects. However, based on the concentration required to active some of these modes of action, only part of these mechanism, seems to be significant in vivo. In pharmacokinetics studies ACR achieved plasma concentration of *ca.* 0.34 μM, however its high distribution to tissues, high cellular accumulation pattern of anthracyclines and formation of active metabolites suggest possible high concentration of the drug in cancer cells [41]. In vitro submicromolar activities were found in case of apoptosis induction, ROS-generation, cells differentiation, chemosentitization and radiosentitization, therefore these effects are expected to be important part of ACR activity in vivo*.*ACR seems to be a particularly promising candidate for combination therapies with other anticancer drugs. This is related to the ACR sensitization of cells to other drugs and the lack of cross-resistance between drugs, as well as, prominent cardiac safety profile. However, promising data form clinical trials require further confirmation. ACR require more, recent, high quality, multicentre clinical trials, involving more participants and control groups for better characterization of its safety and efficacy in specific types of cancer. A problem might be a fact that ACR was discovered and patented over 40 years ago, and pharmaceutical companies may be no interest in development of a drug, whose patent protection has expired. While it routinely used in some countries in Asia (e.g. Japan) its entry to other markets is doubtful [85].

## Data Availability

Data available on request.
